# Spatial patterns of vascular plant species richness in Poland - a data set

**DOI:** 10.1038/s41597-023-02446-y

**Published:** 2023-08-18

**Authors:** Tomasz H. Szymura, Henok Kassa, Grzegorz Swacha, Magdalena Szymura, Adam Zając, Zygmunt Kącki

**Affiliations:** 1 Botanical Garden, University of Wrocław, ul. Sienkiewicza 23, 50-335 Wrocław, Poland; 2 Department of Ecology, Biogeochemistry and Environmental Protection, University of Wrocław, Kanonia 6/8, 50-328 Wrocław, Poland; 3 Institute of Agroecology and Plant Production, Wrocław University of Environmental and Life Sciences, Grunwaldzki Sq. 24A, 50-363 Wrocław, Poland; 4 Institute of Botany, Faculty of Biology and Earth Sciences, Jagiellonian University in Kraków, Kopernika 27, 31-501 Kraków, Poland

**Keywords:** Biodiversity, Biogeography, Macroecology, Invasive species

## Abstract

Recognition of species richness spatial patterns is important for nature conservation and theoretical studies. Inventorying species richness, especially at a larger spatial extent is challenging, thus different data sources are joined and harmonized to obtain a comprehensive data set. Here we present a new data set showing vascular plant species richness in Poland based on a grid of 10 × 10 km squares. The data set was created using data from two sources: the Atlas of Distribution of Vascular Plants in Poland and the Polish Vegetation Database. Using this data set, we analysed 2,160 species with taxonomical nomenclature according to the Euro + Med PlantBase checklist in 3,283 squares covering the entire territory of Poland (ca. 312,000 km^2^). The species were divided into groups according to their status and frequency of distribution, and the statistics for each square were obtained. For purposes of analysis, sampling bias was assessed. The data set promotes theoretical analysis on species richness and reinforces the planning of nature conservations.

## Background & Summary

Knowledge on the spatial patterns of species richness is essential for ecology, biology and nature conservation^1–4^, and it is especially important because of currently accelerating biodiversity loss related to global changes^5^. In the era of big data, merging data from different sources is desirable to obtain comprehensive data sets^3,4,6^. Such a data set needs harmonisation^7^ and bias assessment^8^, and it should be accessible to the public^9^.

Poland is a large country in Central Europe with a land surface area of approximately 312,000 km^2^. The climate is classified as temperate warm transitional^10^, but the territory is crossed by air masses from both the Atlantic Ocean and the heart of the Eurasian landmass, and the continental impact increases gradually from west to east^10^. In the north, the vegetation consists of Baltic Sea coastal habitats, while the south is dominated by the alpine vegetation of the Sudety and Carpathian Mountains. In terms of the biogeographical regions of Europe, Poland is within continental and alpine regions and borders a boreal region^11^. Owing to its geographical location, Poland has various climate types within its borders, leading to the country having boundary ranges of numerous plant species and species representing different floristic elements^12^. Plant studies in Poland have a long tradition. In particular, the phenomenon of range limits of several tree species within Polish territory intrigued early 19th century naturalists^13,14^, and it was continuously studied in a scientific way^15,16^, resulting in the first maps of tree species distribution^17,18^. The classical phytosociological studies started in the 1920s including, among others, steppe vegetation^19^, mountain vegetation^20^, and forests^21^. The knowledge regarding the vegetation of Poland was synthesized in book ‘The Vegetation of Poland’ in 1959^22^, with English edition^23^. However, mapping of vascular plant species richness has never been done in Poland. The primary data source that can be used for such mapping is the ATPOL project – Atlas of Distribution of Vascular Plants in Poland^24–26^. Another recently available data set important for mapping purposes is the Polish Vegetation Database (PVD)^27^. Neither of those projects focused directly on species richness and has not been used for this purpose so far. Here, we present the results of merging and harmonizing the two databases to obtain a comprehensive data set showing the spatial patterns of vascular plant species richness in Poland. The new data set was reinforced by the classification of plants regarding their status in Polish flora. This data set can be used for both biogeographical studies on species richness patterns and for nature conservation purposes.

## Methods

### Original species distribution data and spatial grid

The original data sources recorded the distribution of plants with different taxonomic levels. Mostly, the taxonomic level was species, but subspecies, varietas, species *sensu lato*, aggregations, and hybrids were also included. For simplification, we refer to all of them as ‘species’ if a detailed distinction is not necessary.

**Fig. 1:**
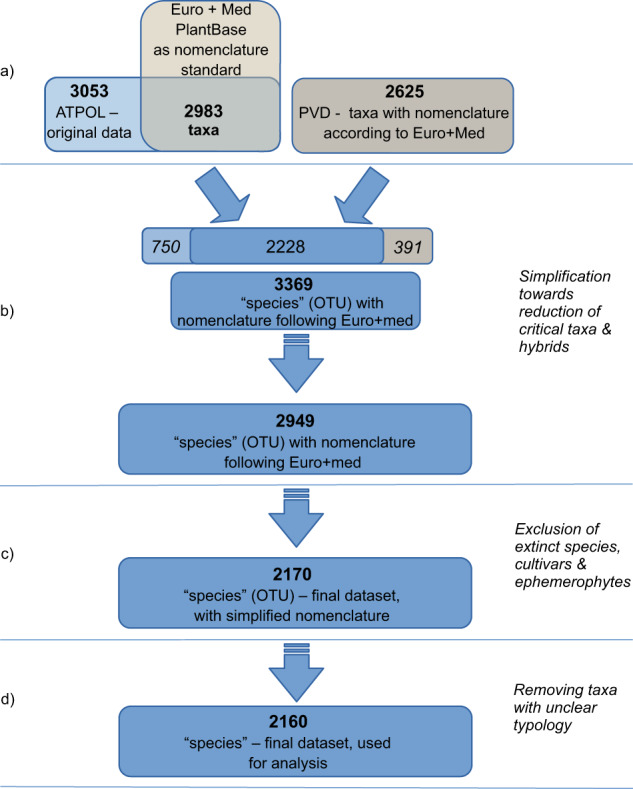
Scheme of harmonisation. (**a**) Standardization of nomenclature following Euro + Med. (**b**) Dataset joining and simplification towards reduction of critical taxa. Among 3,369 species 2,228 were recorded in both data sets, while 750 were contributed exclusively by ATPOL and 391 by PVD. (**c**) Exclusion of extinct species, cultivars and ephemerophytes. (**d**) Removal of taxa with unclear typology.

The ATPOL data were derived from mapping the occurrence of vascular plants, using the cartogram method in 10 × 10 km squares (henceforth, squares). The ATPOL project was launched in the late 1970s by^24^ and is still running. Floristic data of ATPOL contain the code of a square (or the geographical coordinates) and the geographical name of the locality. All available and reliable floristic data in the territory of Poland are being used for ATPOL: results of original field studies and data from literature and herbarium records. The field data can be both a single species occurrence record in a given locality or a list of many species assigned to a locality. To fill a square, it is only necessary to find a single locality of the species inside its area. The taxonomical nomenclature is mostly based on the floristic list published by^28^, but it has been extended as the project has progressed^24^. So far, the project has published two atlases of plant distribution in Poland: the main part was published in 2002^25^ and an appendix followed in 2019^26^. The data contributed by A. Zając, for the purposes of this project, consist of the last version of the ATPOL project (data transferred on 10 November 2020) with information on the distribution of 3,053 plant taxa in 3,283 squares (Fig. 1). The ATPOL project spanned the digital revolution, and the software used for data input, storage and handling has changed over time. Consequently, the number and date of particular records in a square are no longer accessible. The original spatial grid of 10 × 10 km squares has been modified to the recent GIS standards of^29^ and^30^, and for our project, we used the grid system from an online source (https://worldbig.org/atpol/).

**Fig. 2:**
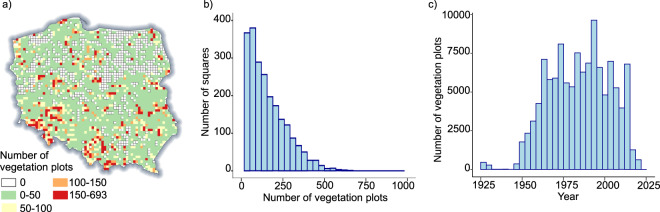
Polish Vegetation Database plot number per square (**a**) and its distribution (**b**) as well as plot recording in years (**c**).

The PVD, which was derived from published and unpublished data for the territory of Poland, was launched in 2007^27^. The PVD stores vegetation plots, including information of species co-occurrences (so-called phytosociological relevés), that are typically collected according to the Central European phytosociological method^31^. Based on the number of plots it contains, the PVD is among the largest vegetation databases in Europe and worldwide^32,33^. The database is registered in the Global Index of Vegetation-Plot Databases (GIVD)^32^ under code EU-PL-001, and it is one of the largest contributors of vegetation data to the European Vegetation Archive^33^ and sPlot^34^. The PVD data consist of 117,328 georeferenced vegetation plots. Data on species occurrences were derived from each vegetation plot based on its georeferenced location and assigned to particular squares. The spatial location of plots is estimated based on plot description (e.g., a particular mountain, forest complex or nearest village) or the coordinates measured using the Global Navigation Satellite System. The data contributed by PVD were obtained on 15 February 2022 and consisted of 117,328 georeferenced vegetation plots, covering the time frame from 1925 to 2020 (Fig. 2). In this project, the species occurrence was extracted from the list of in a plot and the location of the point was assigned to a particular square. From the PVD, we obtained information on the distribution of 2,625 plant taxa in 2,593 squares (Figs. 1, 2).

### Taxonomical harmonisation

**Fig. 3:**
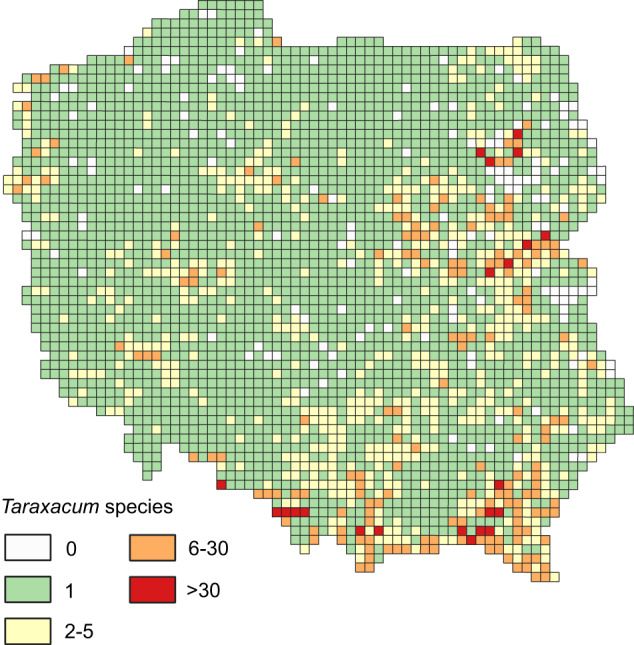
*Taraxacum* species richness distribution.
- Vascular plant species with taxonomical nomenclature that changed over time or those that were difficult to distinguish from one another due to morphological similarity underwent simplification using taxa aggregation (e.g., *Festuca ovina* agg., *Eleocharis palustris* agg.) and *sensu lato* (e.g., *Erigeron acris* s.l.).
- Taxa not recognised at a species level (e.g., hybrids between species, and taxa described as *Crataegus monogyna et laevigata*) were excluded. However, if a hybrid already existed as an aggregation (agg.) of species *sensu lato* (s.l.) and both parental taxa of the hybrid could be included in the already existing aggregation, it was included in the group.

- For the purpose of unification, Euro + Med PlantBase (http://www.europlusmed.org) was used as a common taxonomical nomenclature source. Species considered in Euro + Med as ‘preliminarily accepted’ were also included in the list. Nonetheless, some aggregations and other taxonomical units (e.g., species *sensu lato*) were created as needed. This list of operational taxonomic units (OTUs) was used for further analysis. The application of OTUs allowed retaining some taxa inconsistent with the Euro + Med species list (see points c–e, below), which were further included into an aggregation or other taxonomical unit.
- Cultivars and ephemerophyte species (e.g., *Zea mays* L., *Yucca flaccida* Haw.) were excluded from analysis since the distribution of those species was directly related to human decision-making and was not relevant to ecological problems. Further, species extinct in Poland (e.g., *Cuscuta epilinum* Weihe.) were excluded from the list.
- Six genera (*Alchemilla*, *Hieracium*, *Pilosella*, *Rosa*, *Rubus* and *Taraxacum*) were considered at genus level (e.g., *Taraxacum* sp.) because they consist of species difficult to identify at the species level (so-called microspecies^35,36^) or their taxonomical status changed over time. Consequently, the knowledge regarding the distribution of species within these genera is fragmentary and usually limited to areas surveyed by a taxonomist specialising in particular genera. An example is the distribution of species within *Taraxacum* (Fig. 3) for which 286 taxa (mostly species) were identified in both databases. However, in some squares the number of species recorded was above 30, while only one species was recorded in neighbouring squares with similar environment conditions (Fig. 3), which seems unlikely.

The procedures of taxonomic harmonisation caused loss of some information. Some taxa reported in ATPOL were included in others after application Euro + Med nomenclature instead of the project’s original checklist (Fig. 1). Thus, the number of taxa originally recorded in ATPOL was reduced from 3,053 to 2,983. In addition, the simplification after merging ATPOL and PVD caused the number of species under consideration to decrease by 420; however, the ‘lost’ species were mostly within five genera: *Alchemilla*, *Hieracium*, *Pilosella*, *Rubus*, and *Taraxacum*, with *Taraxacum* alone initially being represented by 286 species.

### Taxa classification

The species were classified according to their affinity to taxonomic units (family, genera), status in Polish flora (native, archeophytes, neophytes), conservation status (Red List species), and frequency of their distribution (rare, moderate and common). The status of species (native, archeophytes and neophytes) was checked according to^37^. The archeophytes, as species with specific ecology and biology, among which some are considered to have high conservation value, were considered as native taxa in the analysis, thus only the neophytes were considered as alien. The species with high conservation value were distinguished based on the Polish Red List^38^. Additionally, we classified native species which occupy human made habitats as apophytes. The apophytes were checked based on an unpublished list provided by A. Zając. The frequency distribution classes are represented by three categories: common, moderate and rare. Common species are those species present in more than 75% of the total number of squares (3,283 squares), moderate species are those present in between 25% and 75%, and rare species are found in less than 25% of the total squares.

In the case of species aggregation or species *sensu lato*, the taxa within the group could represent different affinities towards their status (i.e., native or neophytes) and conservation value (i.e., Red List). In such a case, the rules of classification were the following:

- If a species is present on the Red List, all subspecies belonging the species are also considered as Red List taxa.
- If aggregation or species *sensu lato* consist of two or more taxa, and if all the taxa are considered as Red List, the entire aggregation is considered as Red List.
- If no more than 5% of species in a group represented different status/conservation value or if the taxa occurred rarely (less than 5% of all squares where taxa belonging to the aggregation were found), their presence was ignored and the entire group was classified according to the dominant category. For example, *Diantus superbus* aggregation consisting of *D*. *speciosus* and *D. superbus* subsp*. alpestris* was considered as a Red List aggregation because the non-Red List *D. speciosus* is very rare (ca. 1% of squares in the entire aggregation) compared with *D. superbus* subsp*. alpestris*. However, if the situation was opposite (i.e., the Red List taxon was very rare) the entire aggregation was not considered as a Red List aggregation. For example, very rare *D. carhosianorum* subsp. *Saxigenus* was a Red List species, but *D. carthusianorum* was more frequent and not a Red List species, thus the taxon *D. carthosianorum* s.l. was not considered as a Red List species.

Additionally, we also excluded some aggregations and genera from the joint data set before analysis because of a status problem: Species present in OTUs as both alien and native exceeding 5% of the squares in number hindered categorization of the group as either native or neophyte. In such a case, the simplification considerably influenced the calculated fraction of neophytes in the square. This case included two genera: *Hieracium* and *Rosa*. The same decision was made for the following taxa: *Amaranthus hybridus* agg., *Chenopodium album* agg., *Gentianella campestris* s. l., *Gentianella germanica* s. l., *Laserpitium krapfii* subsp*. krapfii*, *Oenothera biennis* agg., *Onobrychis viciifolia* agg. and *Polygala chamaebuxus*.

### Methods of the data set overview

The final list used for analysis and mapping was based on OTUs, and thus, it included taxa at different taxonomical levels (Fig. 1). For simplification, we considered all the OTUs as species, and for the results, we refer to ‘species richness’. Since the observed species richness is correlated with sampling area, we decided to exclude squares placed partially outside the territory of Poland for dataset analysis and visualisation. We decided to consider only squares with more than 80% of area within the terrestrial territory of Poland; nonetheless, data for all squares are stored in the dataset^39^. A total of 268 cross-boundary squares were excluded because of their location, which consisted of 8% of all analysed squares.

**Fig. 4:**
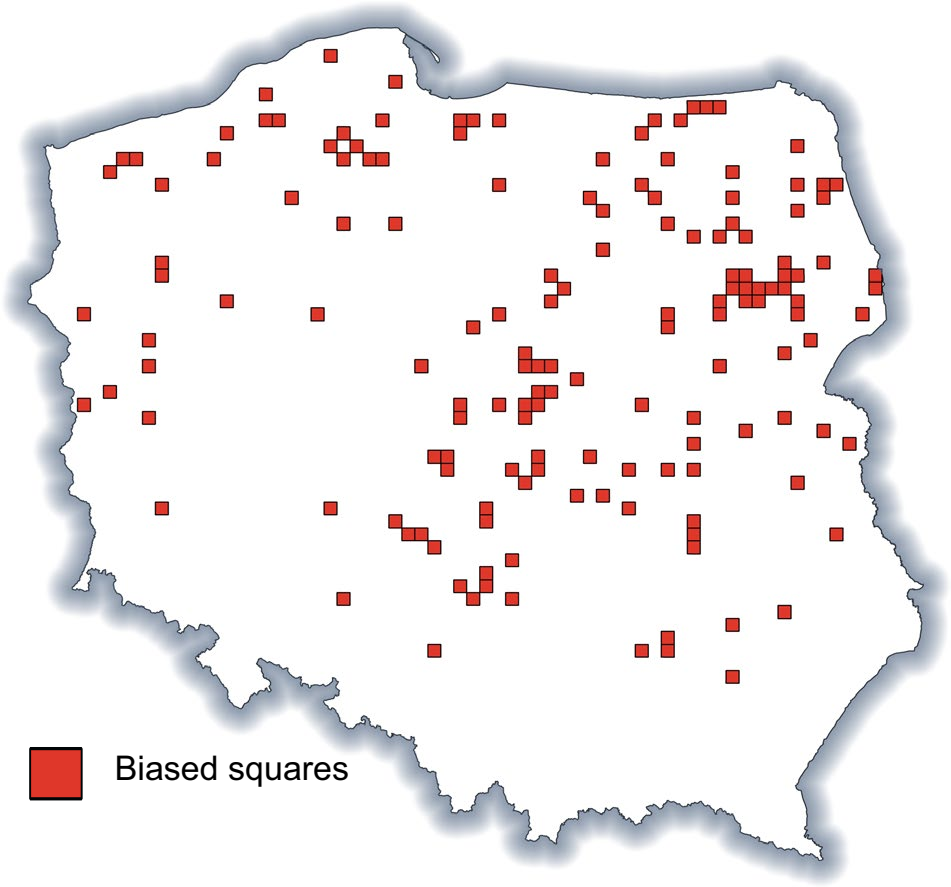
Location of squares considered as undersampled.

In some areas, the sampling effort was very probably low, which in turn, would have affected the species richness estimation. To detect potentially undersampled squares, we employed a simple procedure: The 20 most frequent species in the dataset were determined, and then the species were checked for their geographical ranges and ecological niche. Since the top 20 frequent species were found over the entire territory of Poland and are common species, we considered them as a ‘wish’ list of species which should be recorded in each square. Next, we searched for squares where three or more species from the wish list were missing, and those squares were considered as undersampled and removed from the analysis. The procedure relied on the assumption that if no data were collected from a square for several species from this group, other species were most probably also omitted from the inventory. An analogous basic assumption applied by Kühn *et al*.^40^, relied under the benchmark species approach^41,42^ and for producing biogeographical ignorance maps^43^. The applied procedure resulted in the identification of 149 potentially undersampled (low sampling effort) squares, which consisted of 5% of all analysed squares (Fig. 4).

The applied exclusion criteria changed the species richness in squares, as shown in the statistic result of different exclusion criteria under Table 1.

**Table 1:** Basic statistics for entire data set, after exclusion of cross-boundary squares, and after exclusion of both cross-boundary as well as biased squares (clean data set).

	Entire data set	Without cross-boundary squares	Clean data set (cross-boundary and potentially biased squares excluded)
Number of 10 × 10 km squares	3,283	3,071	2,866
Joined species richness	2,160	2,137	2,137
Average joined species richness per square	496	504	522
Min-max species richness per square	0–1,114	21–1,114	119–1,114

## Data Records

The data set is available at Zenodo repository^39^ under a Creative Commons Attribution 4.0 International licence. This dataset consists of 5 files (Files_description, Taxa_list, Taxa_status, Species_richness and Map_data):

**Files_description -** file with a description of the data stored.

**Taxa_list**. List of taxa. The nomenclature according to Euro + Med PlantBase (Euro + Med.) and operational taxonomical units (OTUs) used for analysis and mapping in the project. For simplification, the taxonomical operational units are called ‘species’.

**Taxa_status**. The species affinity to taxonomic units (family, genera), status in Polish flora (native, archeophytes, neophytes), conservation status (Red List species), and frequency of their distribution (rare, moderate and common). The status (native, archeophytes and neophytes) was checked according to^37^, the high conservation value according to Polish Red List^38^, and the apophytes according to an unpublished list provided by A. Zając. Common species are those species present in more than 75% of the total number of squares (3,283 squares), moderate species are those present in between 25% and 75%, and rare species are found in less than 25% of the total squares.

**Species_richness**. Statistics on species richness and frequency in species groups for 10 × 10 km ATPOL squares. The names of squares according to original names in the ATPOL project^24^. The sampling bias (SB) shows adequately sampled squares labelled with 1, while squares with 0 are those with low sampling effort. Cross-boundary squares (CBS) denoted by 1 are squares with more than 80% of the area within the terrestrial territory of Poland, while squares with CBS of 0 are those with 80% or less of the area within the terrestrial territory of Poland. The detail information about the particular columns is shown in ‘Files_description’ and ‘Taxa_status’ files.

**Map_data**. A shapefile with squares geospatial locations, codes of their names, and data on species richness and frequency in species groups. The map is registered in WGS 84 coordinate reference system (EPSG code 4326). The abbreviations and square names used in ‘dbf’ file are the same as those used in ‘Species_richness’ file.

Abbreviations used in the dataset are explained in Table 2.

**Table 2:** Table with explanations of abbreviations used in the original dataset.

Abbreviations	Name	Description
Square_Id	Id of ATPOL 10 × 10 km square	Original nomenclature used in ATPOL project
JSR	Joined species richness	The species merged from PVD and ATPOL
NaS	Native species	
NaS_‘%’	Percentage of native species	NaS_‘%’ = (NaS /JSR)*100
ReLS	Red List species	Species with high conservation value in Poland
ReLS_‘%’	Percentage of Red List species	ReLS_‘%’ = (ReLS/JSR)*100
NeS	Neophyte species	
NeS_‘%’	Percentage of neophyte species	NeS_‘%’ = (NeS /JSR)*100
ArS	Archeophyte species	
ArS_‘%’	Percentage of archeophyte species	ArS_‘%’ = (ArS /JSR)*100
‘Ar + Na’S	Archeophyte and native species	Because the archeophytes are a species with specific ecology and biology, among which some are considered as high conservation value, they can be included in the calculation of native species richness
‘Ar + Na’S_‘%’	Percentage of archeophyte and native species	‘Ar + Na’S_‘%’ = (‘Ar + Na’S /JSR)*100
ApS	Apophyte species	
ApS_‘%’	Percentage of apophyte species	ApS_‘%’ = (ApS /JSR)*100
RS	Rare species	Rare species are species present in less than 25% of the total squares (3,283 squares)
RS_‘%’	Percentage of rare species	RS_‘%’ = (RS /JSR)*100
MSR	Moderate species richness	Moderate species are species present in between 25% and 75% of the total squares (3,283 squares).
MS_‘%’	Percentage of moderate species	MS_‘%’ = (MS/JSR)*100
CS	Common species	Common species are those species present in more than 75% of the total squares (3,283 squares).
CS_‘%’	Percentage of common species	CS_‘%’ = (CS/JSR)*100
NG	Number of plant genera	Number of genera represented by species in a particular square
NF	Number of plant families	Number of families represented by species in a particular square
SB	Sampling bias	Squares with 1 are adequately sampled squares, while squares with 0 have low sampling effort.
CBS	Cross-boundary squares	Squares with 1 are squares with more than 80% of area within the terrestrial territory of Poland, while squares with 0 are those with 80% or less of area within the territory.

## Technical Validation

**Fig. 5:**
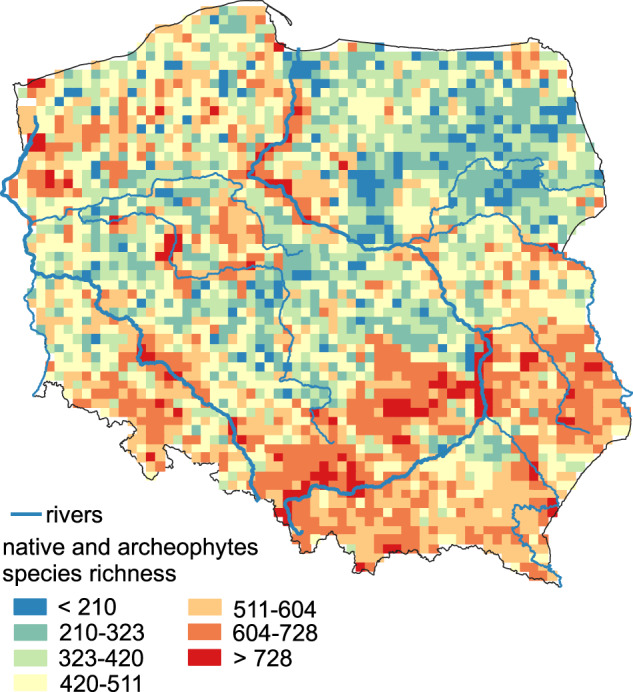
Native and archeophyte species richness.

The dataset is stored in simple formats (xlsx and shp). The data were already used for preparing scientific articles (submitted) and for calculating statistics presented at scientific conferences, which confirms that the data set is functional using typical software for data analysis/visualisation (e.g., Fig. 5).

## Usage Note

1. For visualisation, we suggest using only data for squares with more than 80% of area within the terrestrial territory of Poland. The recommended squares are denoted by value 1 in column CBS in ‘Species_richness’ file (for details see ‘Methods of the data set overview’). The map for native and archeophyte species richness is shown on Fig. 5).
2. For statistical analysis, we suggest excluding squares that are potentially undersampled (for details see ‘Methods of the data set overview’). The squares are denoted by value 1 in column SB in ‘Species_richness’ file. We also suggest excluding squares mentioned in point 1 from analysis.
3. This dataset can be temporally biased: It predominantly reflects the species richness pattern from 1960 to 2000s since most of field data comes from this period. Unfortunately, we did not have field data or models for assessing changes of species richness caused by extinction of species within a square. Regarding the alien species, the ATPOL data were upgraded in 2019^26^.
4. The species richness patterns will change as new data are added, species become extinct, and taxonomical approach and species classification change (e.g., changes in Red List, naturalisation of ephemerophytes). Therefore, we consider the presented data set as version 1.1, designed for further development and actualisation.

## Data Availability

No specific code was used to produce and analyse the presented data.
